# ﻿Two new species of the planthopper genus *Usana* Distant, 1906 (Hemiptera, Fulgoromorpha, Achilidae) from China

**DOI:** 10.3897/zookeys.1184.103943

**Published:** 2023-11-23

**Authors:** Xiu-Dong Huang, Lin Yang, Xiang-Sheng Chen, Jian-Kun Long

**Affiliations:** 1 Institute of Entomology, Guizhou University, Guiyang, Guizhou 550025, China; 2 The Provincial Special Key Laboratory for Development and Utilization of Insect Resources of Guizhou, Guizhou University, Guiyang, Guizhou 550025, China; 3 Anshun Academy of Agricultural Sciences, Anshun, Guizhou 561000, China

**Keywords:** Achilidae, distribution, Fulgoromorpha, taxonomy

## Abstract

Two new species of the achilid planthopper genus *Usana* Distant, 1906 (Hemiptera, Fulgoromorpha, Achilidae, Myconinae, Plectoderini), *U.tongmaiensis* Long & Huang, **sp. nov.** and *U.rotalarius* Long & Huang, **sp. nov.**, are described and illustrated from Xizang and Chongqing. A key to all known species and a map of geographic distributions for Chinese taxa is provided.

## ﻿Introduction

Achilidae Stål, 1866 constitutes one of the moderate-sized families within planthoppers (Hemiptera, Fulgoromorpha) with 521 described species in 162 genera and three subfamilies: Apatesoninae Metcalf, 1938, Achilinae Stål, 1866, and Myconinae Fennah, 1950 (Bartlett et al. 2018; Bourgoin 2023); only the last two only subfamilies occur in China. Plectoderini is the richest tribe in Achilidae, with 353 species and 99 genera worldwide, of which 17 genera and 80 species occur in China. *Usana* Distant, 1906 is a small genus of planthoppers in the tribe Plectoderini with 10 species. It has recently been reviewed (Long et al. 2015). It comprises only 2.8% of known Plectoderini diversity (Bourgoin 2023).

*Usana* was first described by Distant, with *U.lineolalis* Distant, 1906 from Burma as its type species (Distant 1906). Ten species are now known in the genus: *Usanalineolalis* Distant, 1906; *U.abdominalis* Distant, 1916; *U.aspergilliformis* Long, Yang & Chen, 2015; *U.concava* Long, Yang & Chen, 2015; *U.congjiangensis* Long, Yang & Chen, 2015; *U.demochares* Fennah, 1978; *U.fissura* Long, Yang & Chen, 2015; *U.oblongincisa* Long, Yang & Chen, 2015; *U.unispina* Long, Yang & Chen, 2015; and *U.yanonis* Matsumura, 1914 (Bourgoin 2023).

Recent study of some Chinese *Usana* specimens has revealed two new species, described here as *U.tongmaiensis* Long & Huang, sp. nov. and *U.rotalarius* Long & Huang, sp. nov. Including these new species, the genus currently now has 12 species distributed in the Palaearctic, Oriental, and Sino-Japanese realms.

## ﻿Materials and methods

### ﻿Materials

Type materials are deposited in the Institute of Entomology, Guizhou University, Guiyang, Guizhou Province China (**IEGU**).

### ﻿Preparations and illustration

The colour photographs were taken using a Canon 5D Mark IV camera in conjunction with a Canon EF 100 mm F/5.6L IS USM macro lens, and a Godox MF12 macro flash 2-light kit was used as light source. Zerene Stacker v. 1.04 was used for focus stacking. External morphology was observed under an Olympus SZX7 stereoscopic microscope. Measurements were made with the aid of a Keyence VHX-1000E system. The genital segments of the examined specimens were macerated in 10% KOH, then transferred to glycerol for examination. Drawings and external morphology were done with the aid of a Leica MZ 12.5 stereo microscope. Illustrations were scanned by a Canon CanoScan LiDE100 and imported into Adobe Photoshop CS6 for labeling and plate composition.

### ﻿Measurements and abbreviations

**Body length** length of specimen from apex of vertex to fore wing apex (in dorsal view);

**A/B** width of vertex at posterior margin / length of vertex at midline;

**C/D** length of frons at midline / maximum width of frons;

**D/E** maximum width of frons / width of frons at apex;

**F/C** length of postclypeus at midline / length of frons at midline;

**G/H** length of apical / length of subapical;

**I/B** length of pronotum at midline / length of vertex at midline;

**J/I** length of mesonotum at midline / length of pronotum at midline;

**J/B+I** length of mesonotum at midline / cumulative length of vertex and pronotum at midline;

**K/L** length of fore wing from the base to the apical margin in median portion / width of fore wing at the widest part;

**M/N** length of hind wing from the base to the apical margin in median portion / width of hind wing at the widest part.

### ﻿Terminology

The nomenclature of the wing veins follows the interpretation proposed by Asche (2015) and Bourgoin et al. (2015). The morphological terminology and measurements used in this study follow Chen et al. (1989) and Yang and Chang (2000). The zoogeographic regions employed in this study adhere to the classification proposed by Holt et al. (2013).

## ﻿Taxonomy

**Family Achilidae Stål, 1866**

**Subfamily Myconinae Fennah, 1950**

### ﻿Tribe Plectoderini Stål, 1950

#### ﻿ Usana

Taxon classificationAnimaliaHemipteraAchilidae

Distant, 1906

9FD0136E-EADD-55FD-BA5F-C6C34C7D6087

Usana Distant, 1906: 293; Fennah 1950: 132; Fennah 1978: 249; Chen et al. 1989: 23.

##### Type species.

*Usanalineolalis* Distant, 1906, by original designation.

##### Diagnosis.

***Head*.** Width of head at eyes 0.8–0.9 times wider than pronotum. Vertex not declivous, broader at base than long in middle line, median carina distinct, anterior margin carinate, triangular areolets at lateroapical angles of head distinct, posterior margin broadly concave. Frons longer at mid-line than at widest part, basal margin truncate, median carina distinct, lateral margins carinate. Rostrum almost or just reaching post-trochanter, with subapical segment shorter than apex.

***Thorax*.** Pronotum lateral lobe not inclined anteroventrally, with a small longitudinal carina between eye and tegula. Mesonotum length at midline longer than vertex and pronotum combined. Fore-wing longer than widest part by ~2.7–3.3: 1; vein ScP+R with fork distally or as level of CuA fork; vein MP with fork clearly after CuA fork, with 3 terminals. Hind wing with MP with 3 terminals (MP_1_, MP_2_ and MP_3+4_), vein CuA with 2 terminals. Post-tibiae with a lateral spine between basal 1/3 to near basal 1/2, spinal formula 7–7 (6)–7 (6).

***Male terminalia*.** Length of anal segment in dorsal view at least equal to its width; apical margin of anal segment distinctly excavated at midline; pygofer in lateral view with dorsal margin distinctly shorter than ventral margin, medioventral process entire or with apex divided into 2 branches. Genital style with 3 processes arising from its dorsal margin, inner surface near anterior margin with a long, outwardly directed process. Phallobase sheathed, generally asymmetrical, with apical 1/2 divided into a dorsal, 2 lateral, and a ventral lobe: dorsal lobe relatively short, lateral lobes valviform, and ventral lobe with apical margin incised at midline and with subapical surface in middle giving rise to a long process, directed basad. Each phallic appendage generally not exceeding apical margin of phallobase, with a protrusion between basal 1/4 to 1/3.

##### Distribution.

Palaearctic, Oriental, and Sino-Japanese realms.

### ﻿Checklist and distributions of species of *Usana* Distant, 1906

*U.abdominalis* Distant, 1916; Sikkim.

*U.aspergilliformis* Long, Yang & Chen, 2015; China (Guizhou).

*U.concava* Long, Yang & Chen, 2015; China (Yunnan).

*U.congjiangensis* Long, Yang & Chen, 2015; China (Guizhou).

*U.demochares* Fennah, 1978; Vietnam (Ninh Binh).

*U.fissura* Long, Yang & Chen, 2015; China (Guizhou).

*U.lineolalis* Distant, 1906; China (Jiangsu, Zhejiang, Guangdong, and Guizhou), Burma (Tenasserim and Myitta).

*U.oblongincisa* Long, Yang & Chen, 2015; China (Guizhou, Guangxi, and Hainan).

*U.rotalarius* Long & Huang, sp. nov.; China (Chongqing).

*U.tongmaiensis* Long & Huang, sp. nov.; China (Xizang).

*U.unispina* Long, Yang & Chen, 2015; China (Fujian, Sichuan, and Guizhou).

*U.yanonis* Matsumura, 1914; China (Taiwan), Japan and Korea.

### ﻿Key to species of the genus *Usana* Distant, 1906

Based on Long et al. 2015.

**Table:** 

1	Frons with marking(s)	**2**
–	Frons without marking	**5**
2	Frons with 2 rounded dark brown markings near the apex (Long et al. 2015: fig. 39); disks of pronotum and mesonotum in dorsal view with 8 and 6 longitudinal dark brown stripes, respectively (Long et al. 2015: figs 5, 37)	***U.concava***
–	Frons with dark brown transverse, pronotum and mesonotum not as above	**3**
3	Frons with 1 dark brown transverse at the end	**4**
–	Frons with 1 dark brown straight band at the proximal base and end (Fig. 10)	***U.tongmaiensis* sp. nov.**
4	Fore-wing with several small, transverse, dark-brown stripes especially in area of clavus (Long et al. 2015: figs 1–4, 28); dorsal lobe of phallobase in dorsal view with apical margin aspergilliform (Long et al. 2015: fig. 35); apex of phallic appendage with 2 small spines on inner side (Long et al. 2015: figs 35, 36)	***U.aspergilliformis***
–	Forewing without transverse stripes in area of clavus (Long et al. 2015: figs 9–12, 64) dorsal lobe of phallobase in dorsal view with apical margin roundly convex, and deeply cleft from base to apical 1/4 in midline (Long et al. 2015: fig. 71); apex of phallic appendage without 2 small spines at inner side (Long et al. 2015: figs 71, 72)	***U.fissura***
5	Vertex with 1 or 2 longitudinal brown stripes	**6**
–	Vertex without brown stripes	**8**
6	Discs of vertex and pronotum with 2 longitudinal stripes between lateral carinae respectively	**7**
–	Discs of vertex and pronotum with only 1 longitudinal stripe between lateral carinae respectively (Long et al. 2015: figs 21, 23, 97)	***U.unispina***
7	Genae with a brown spot below antenna (Chen et al. 1989: fig. 9B); tegulae without a spot (Matsumura 1914: fig. 7a; Chen et al. 1989: fig. 9A; Rahman et al. 2014: fig. 7A, B)	***U.yanonis***
–	Genae without a spot below antenna (Long et al. 2015: figs 14, 16, 74); tegulae with a spot (Long et al. 2015: figs 13–16, 73)	***U.lineolalis***
8	Genae with a brown spot below antenna	**9**
–	Genae without spot below antenna	**10**
9	Pronotum and mesonotum with a broad longitudinal brown stripe behind each eye (Long et al. 2015: figs 7, 8, 49); medioventral process of pygofer with apical margin convex, roundly cleft to apical 1/4 at midline (Long et al. 2015: fig. 56)	***U.congjiangensis***
–	Pronotum and mesonotum behind eyes without stripe (Fennah 1978: fig. 186); medioventral process of pygofer with apical margin truncate, angularly cleft to apical 1/2 in the midline (Fennah 1978: fig. 189)	***U.demochares***
10	Ventral lobe of pronotum with a dark brown spot (Long et al. 2015: figs 18, 20); fore-wing yellowish brown with a black marking at base, costal margin and clavus with a longitudinal pale-yellow stripe respectively (Long et al. 2015: figs 17–20, 88)	***U.oblongincisa***
–	Ventral lobe of pronotum with without a spot and without a longitudinal stripe in clavus	**11**
11	Fore-wing pale, yellowish brown, without a marking at base (Distant 1916: fig. 45)	***U.abdominalis***
–	Tegmen light yellowish brown to dark brown (Figs 3, 4, 32); basal cell deep brown at end (Fig. 32); medioventral process of pygofer in ventral view (Fig. 18) entire, apically convex, and concave at base	***U.rotalarius* sp. nov.**

#### ﻿ Usana tongmaiensis

Taxon classificationAnimaliaHemipteraAchilidae

Long & Huang sp. nov.

DC439ADC-E2CE-588B-8B1E-26378F50E31A

https://zoobank.org/32EEF396-2D6E-46FD-A1E7-100CC07616B9

Figs 1 , 2 , 5–8 , 9–14 , 21–23 , 28 , 29

##### Type materials.

***Holotype***: ♂, China: Xizang, Bomi, Tongmai (30°6'N, 95°4'E), 20 August 2020, light trap, Y.-J. Sui leg.; IEGU. ***Paratypes***: 3 ♂; Tongmai; 30°6'N, 95°4'E; light trap, 20 August 2020, Y.-J. Sui leg.; 2 ♂; Tongmai; 30°6'N, 95°4'E; light trap, 12 August 2017, B. Yan leg.; IEGU.

##### Diagnosis.

This species is similar to *U.fissura* in appearance, but differs from that species in the following: frons with 1 dark-brown, straight band at the proximal base and end; end part of postclypeus dark brown (with a brown marking at each side of median carina in *U.fissura*); length of anal segment in dorsal view (Fig. 13) ~1.7 times as long as width (length to maximum width ratio of 1.0 in *U.fissura*); anal segment in lateral view (Fig. 14) with nearly right-angle bend from base to end (not bent in *U.fissura*); lateral lobe relatively simple (right lateral lobe with a large and a small subapical processes in *U.fissura*).

##### Description.

Body length (from apex of vertex to fore-wing apex): male 5.6–6.2 mm (*n* = 5); fore-wing length: male 4.8–5.2 mm (*n* = 5).

**Figures 1–4.**
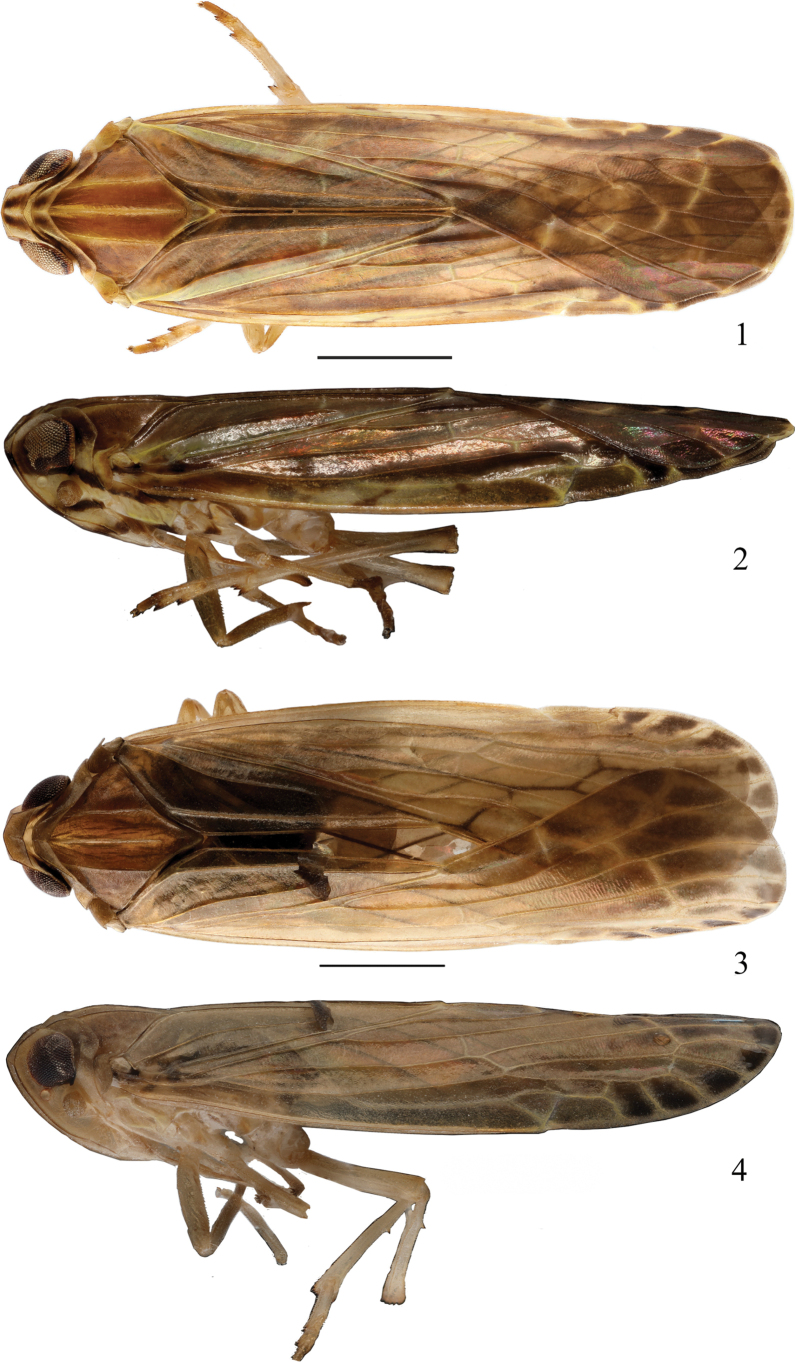
Adult male habitus (dorsal and lateral views) of *Usana* new species **1, 2***U.tongmaiensis* sp. nov. **3, 4***U.rotalarius* sp. nov. Scale bars: 1 mm.

**Figures 5–8.**
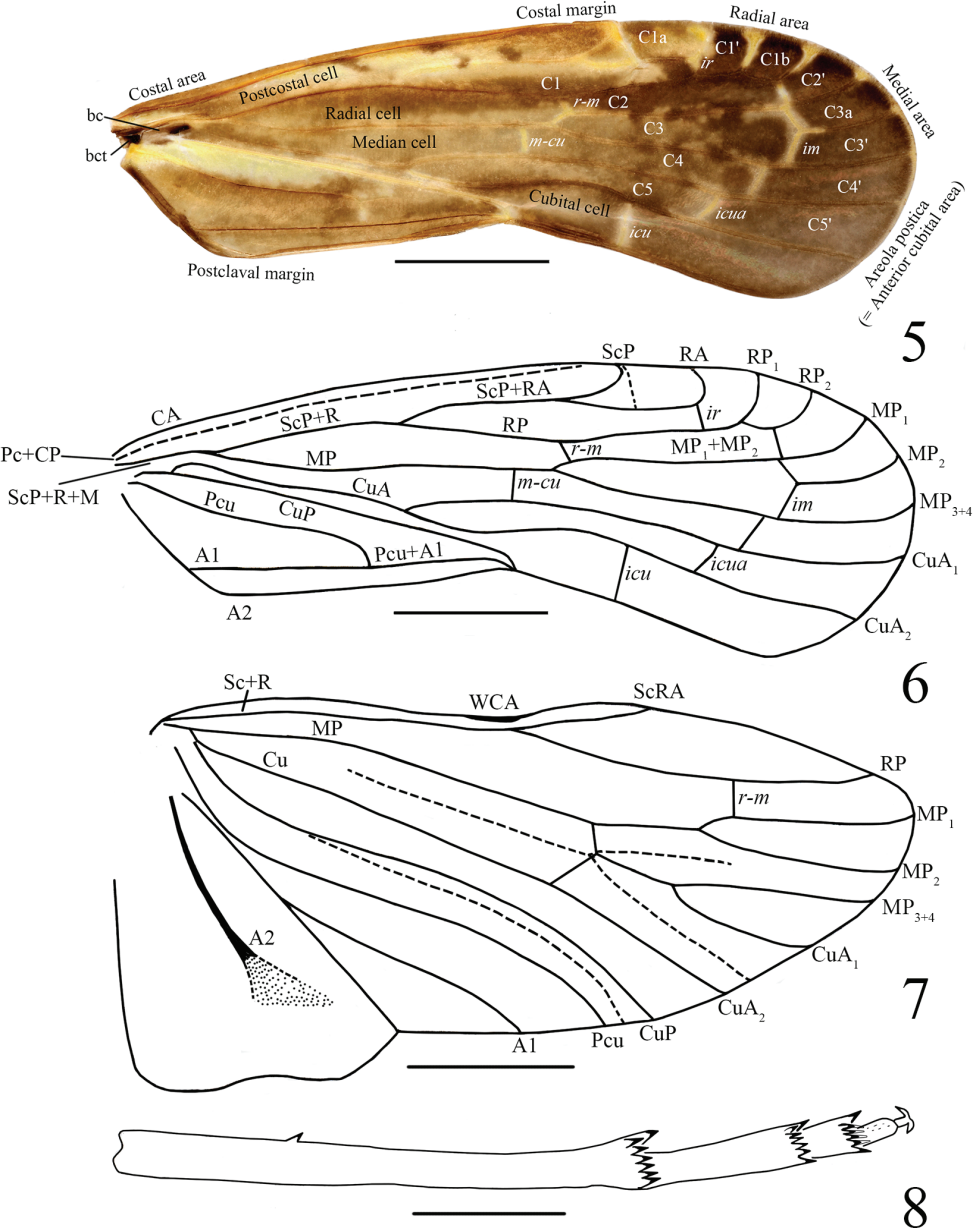
*Usanatongmaiensis* sp. nov., male **5, 6** fore-wing **7** hind wing **8** post tibiae. Scale bars: 1 mm (**5–7**); 0.5 mm (**8**).

**Figures 9–20.**
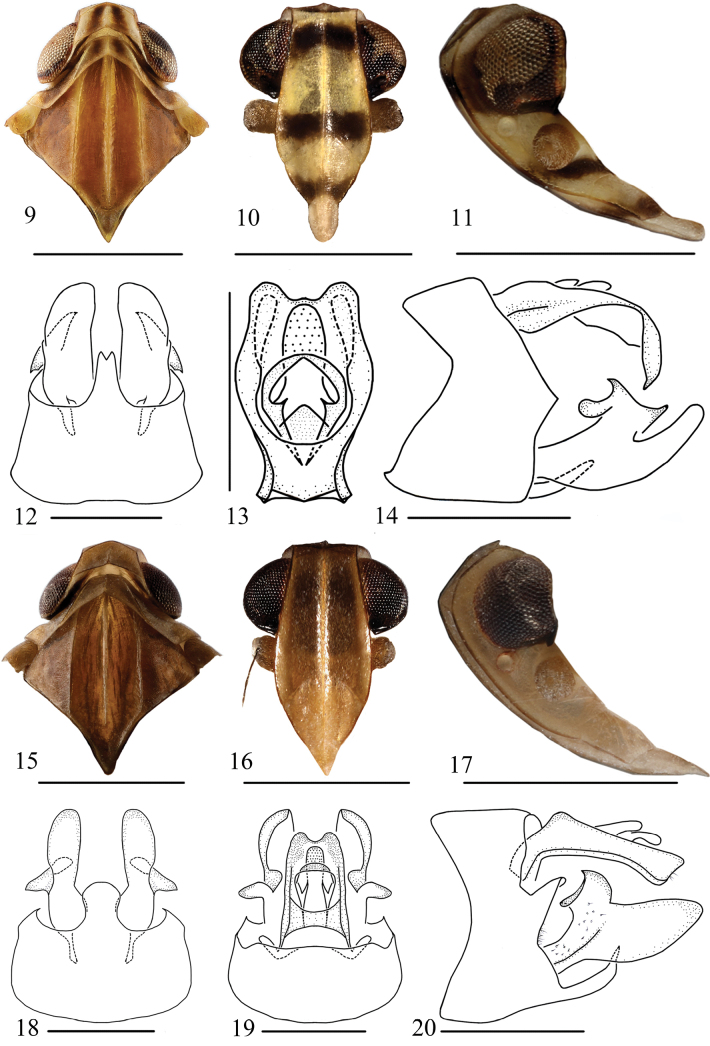
Head, thorax and pygofer of *Usana* new species **9–14***U.tongmaiensis* sp. nov., male **15–20***U.rotalarius* sp. nov., male **9, 15** head and thorax, dorsal view **10, 16** face **11, 17** head, lateral view **12, 18** pygofer and genital style, ventral view **13, 19** anal segment, dorsal view **14, 20** male genitalia, lateral view. Scale bars: 1 mm (**9–11, 15–17**); 0.5 mm (**12–14, 18–20**).

***Colouration*.** Generally yellowish white to dark brown (Figs 1, 2). Vertex yellowish white with 2 longitudinal, dark-brown stripes along midline (Figs 1, 9). Face yellowish white; frons with 1 dark-brown, straight band at proximal base and end; end part of postclypeus dark brown (Fig. 10). Genae yellowish white, with a brown band in front of compound eyes; eyes reddish brown, ocellus yellowish white; Antenna yellowish brown, with 1 dark-brown, oblique stripe below it (Fig. 11). Rostrum yellowish brown, with end brown. Pronotum yellowish brown with 2 dark-brown, longitudinal stripes between lateral carinae, lateral lobe brown behind eyes, and ventral lobe with a longitudinal brown stripe (Figs 1, 2, 9). Mesonotum brown, with middle and lateral ridges yellowish brown (Figs 1, 9). Tegulae with inner half yellowish white, external half yellowish brown (Fig. 9). Tegmen yellowish white to dark brown (Figs 1, 2, 5). Costal area yellowish brown. Postcostal cell yellowish white to brown, with brown near base and on end areas, medial area with 3 irregular, dark-brown spots. Radial area yellowish brown to dark brown; C1a yellowish brown; C1’ and C1b dark brown. Radial cell yellowish brown to dark brown; C2 and C2’ dark brown. Medial area yellowish brown to dark brown; C3 with irregular, deep-brown markings; C3a and C3’ dark brown. Median cell yellowish brown to dark brown; C4 with irregular, deep-brown markings; C4’ dark brown. Areola postica yellowish brown to dark brown; C5’ dark brown. Cubital cell brown to deep brown, with 2 yellowish-white markings at near the middle. Basal cell end half deep brown. Area between CuP and Pcu with base half yellowish white and half brown. Area between Pcu and wing margin brown, with base yellowish white. Hind wing pale brown; veins brown. Legs and abdomen yellowish brown (Fig. 2).

***Head and thorax*.** Vertex not concave, slightly declivous; triangular areolets at lateroapical angles of head distinct; anterior margins carinate; arcus convex forward; lateral margins carinate, relatively straight, and diverging basad; posterior margin broadly concave (Figs 1, 9). Frons slightly convex in lateral view; upper margin (apex) truncate; median carina evident; lateral margin carinate, sinuately diverging to level of antennae, thence gradually incurved to suture (Fig. 10). Clypeus with distinct median and lateral carinae (Fig. 10). Rostrum just reaching trochanter of hind legs (Fig. 2). Antenna nearly cylindrical, not sunken as a depression (Figs 2, 10, 11). Ocelli separated from eyes (Fig. 11). Pronotum with 3 distinct carinae; anterior margin of disk broadly convex; posterior margin obtusely angled, concave at middle; median carina distinct; lateral carinae straight, slightly diverging rearward, attaining hind margin; lateral lobe with a small longitudinal carina between eye and tegula (Figs 1, 9). Mesonotum wider, with 3 obvious, nearly parallel carinae (Figs 1, 9).

Tegmen with costal margin slightly convex; apical margin roundly convex; distinctly concave on postclaval margin (Figs 5, 6). Stem ScP+R+MP short after basal cell before MP fork; stem ScP+R forked at ~1/3 of tegmen length, slightly before CuA fork; vein ScP+RA with fork clearly before RP fork, with 2 terminals; branch RP with 2 terminals; vein MP with fork plainly after CuA fork, with 3 terminals; branch CuA with 2 terminals (Figs 5, 6). Hind wing with simple ScP+RA, branch RP vein with 1 terminal, MP with 3 terminals (MP_1_, MP_2_ and MP_3+4_), vein CuA with 2 terminals, vein A2 without blind branches (Fig. 7).

***Legs*.** Post-tibiae with a lateral spine at basal 1/4. Metatibia with 6 or 7 apical teeth; rightmost apical teeth obviously larger, arrangement slightly slanting; basimetatarsomere with row of 7 apical teeth, their arrangement slightly slanting; midmetarsomere with 5 or 6 apical teeth, their arrangement slightly slanting, each tooth with platellae except for marginal ones, internal spines; metatibio-tarsal formula 7(6)–7–6 (5) (Fig. 8).

***Head*.** Vertex: A/B = 2.3. Frons: C/D = 1.2; D/E = 1.5; F/C = 0.5. Rostrum: G/H = 1.5.

***Thorax*.** Pronotum: I/B = 0.7. Mesonotum: J/I = 9.1; J/B+I = 3.8. Fore-wing: K/L = 3.1. Hind wing: M/N = 1.9.

**Figures 21–31.**
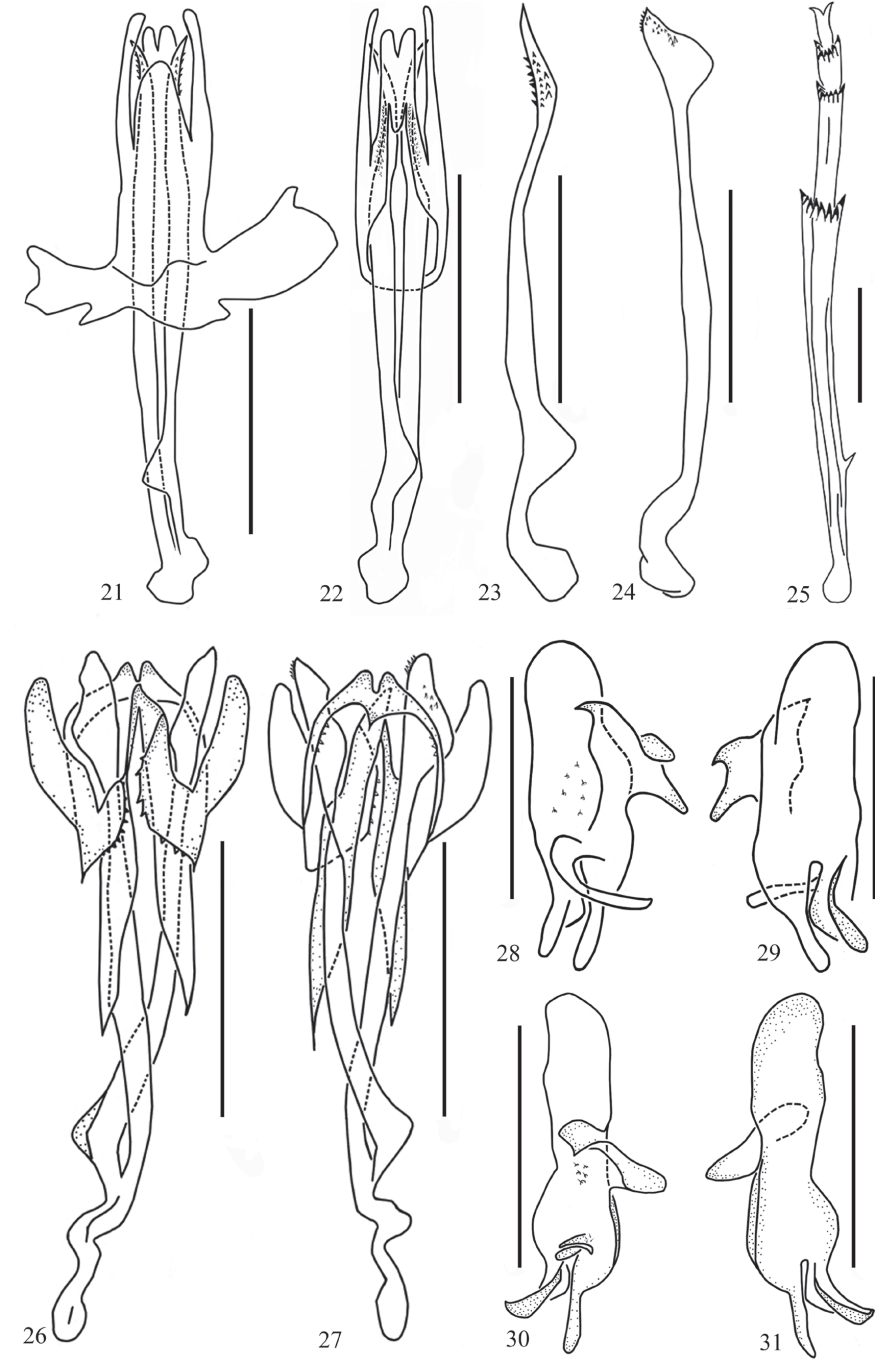
Genital, genital style and post tibiae of *Usana* new species **21–23, 28, 29***U.tongmaiensis* sp. nov. **24–27, 30, 31***U.rotalarius* sp. nov. **28, 30** left genital style, inner surface view **29, 31** left genital style, ventral view **21, 26** aedeagus, dorsal view **22, 27** aedeagus, ventral view **23, 24** inner penis rods in dorsal view **25** post tibiae. Scale bars: 0.5 mm.

***Male terminalia*.** Anal segment in dorsal view (Fig. 13) with maximum width near middle, hence narrowing basad and apically; apical margin roundly concave at middle, length of anal segment ~1.7 times as long as width; anal style not exceeding apical margin of anal segment. Anal segment in lateral view (Fig. 14) with nearly right-angle bend from base to end. Pygofer in lateral view (Fig. 14) with dorsal margin distinctly shorter than ventral margin, anterior margin deep concave at 1/3 its length; posterior margin near middle obviously serrated, convex. Medioventral process of pygofer in ventral view (Fig. 12) entire, apically narrowed, with apical margin angularly incised. Genital style slightly narrowing apically, with apex roundly convex; dorsal margin gives rise to 2 large, sharp processes and 1 short, blunt process; inner surface near anterior margin with a slender, outwardly directed, finger-like process (Figs 12, 28, 29). Aedeagus structure relatively simple, nearly symmetrical, nested (Figs 21, 22). Lateral periandrial lobe of phallobase obviously longer than dorsal and ventral periandrial lobe (Figs 21, 22). Dorsal periandrial lobe unpaired, in dorsal view finger-like (Fig. 21); left and right lateral periandrial lobes nearly symmetrical, with apical margins roundly convex. Ventral periandrial lobe in ventral view (Fig. 22) along inner margin of each lateral side from subapical to middle with a longitudinal group of teeth, subapical surface in middle gives rise to a long process, directed basad, with its apical margin roundly convex. Inner penis rods elongate, lanceolate, curved, apically pointed, basally broadly fused together (Fig. 23).

##### Etymology.

The species name refers to the type locality, Tongmai, Xizang, China.

##### Host plant.

Unknown.

##### Distribution.

China (Xizang).

#### ﻿ Usana rotalarius

Taxon classificationAnimaliaHemipteraAchilidae

Long & Huang sp. nov.

DE46A3F6-C4B0-5D2D-9DA8-C0FEF59691E3

https://zoobank.org/B8B2E0D6-A1EE-4F5C-887F-FA18FE3CB67A

Figs 3 , 4 , 15–20 , 24–27 , 30–31 , 32–34

##### Type materials.

***Holotype***: China • ♂; Chongqing Municipality, Wuxi County, Yintiaoling National Natural Reserve; 31°27'N, 109°56'E; sweeping, 11 August 2022, W.-J. Cao leg.; IEGU. ***Paratypes*** China • 4 ♂; Yintiaoling National Natural Reserve; 31°27'N, 109°56'E; sweeping, 11 August 2022, W.-J. Cao leg.; IEGU.

##### Diagnosis.

The salient features of this new species different from other species in *Usana* are as follows: 1) anal segment in lateral view (Fig. 20) with nearly right-angle bend from base to end; 2) medioventral process of pygofer in ventral view (Fig. 18) entire, apically convex, concave at base; 3) dorsal periandrial lobe in ventral view horseshoe-shaped (Fig. 27), inner margin middle with a longitudinal group of teeth, subapical surface in middle gives rise to a short process with its apical margin sharp convex, directed based; 4) inner penis rods elongate, blade-like, curved, apically pointed (Fig. 24).

##### Description.

Body length (from apex of vertex to fore-wing apex): male 5.6–6.1 mm (*n* = 5); fore-wing length: male 4.8–5.2 mm (*n* = 5).

**Figures 32–34.**
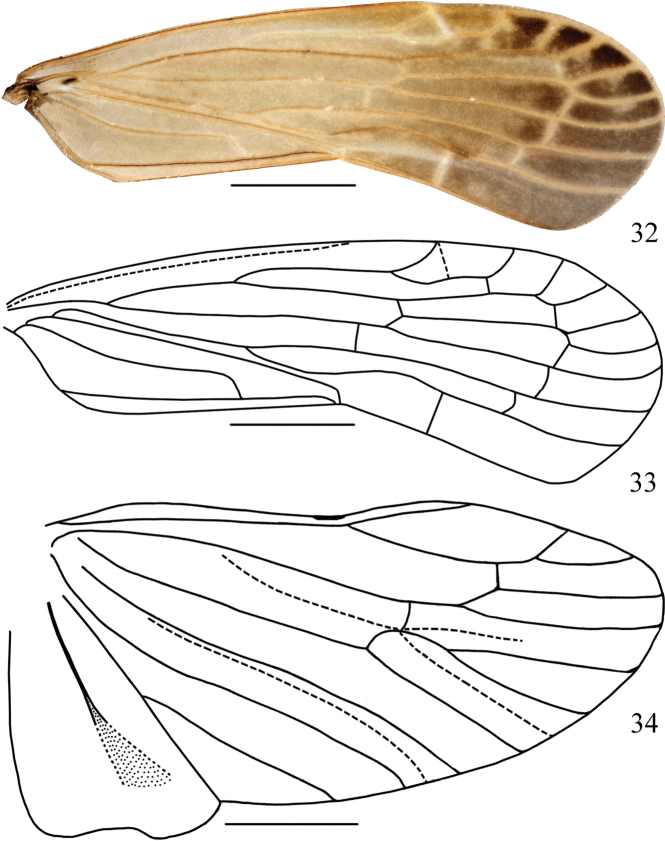
Adult male *Usanarotalarius* sp. nov. **32, 33** fore-wing **34** hind wing. Scale bars: 1 mm.

***Colouration*.** Generally light, yellowish brown to dark brown (Figs 3, 4). Vertex yellowish brown (Figs 3, 15). Face yellowish brown to dark brown; frons brown dark brown on proximal part; postclypeus yellowish brown (Fig. 16). Genae light yellowish brown (Fig. 16). Eyes reddish brown, ocellus light yellowish brown (Figs 16, 17). Antenna yellowish brown (Figs 16, 17). Rostrum yellowish brown, with brown end (Fig. 4). Pronotum yellowish brown (Figs 3, 15). Mesonotum brown (Figs 3, 15). Tegulae yellowish brown (Fig. 15). Tegmen light yellowish brown to dark brown (Figs 3, 4, 32). Costal area yellowish brown. Postcostal cell light yellowish brown to yellowish brown, with end areas yellowish brown. Radial area light yellowish brown to dark brown; C1 light yellowish brown; C1a yellowish brown; C1’ and C1b dark brown. Radial cell light yellowish brown to dark brown; C2 yellowish brown; C2’ dark brown. Medial area yellowish brown to dark brown; C3 yellowish brown, with deep-brown ends; C3a and C3’ dark brown. Median cell light yellowish brown; C4 yellowish brown with end areas deep brown; C4a and C4’ dark brown. Areola postica light yellowish brown to dark brown; C5 base light yellowish brown and end deep brown; C5’ dark brown. Cubital cell yellowish brown to deep brown, with base yellowish brown and end deep brown. Basal cell deep brown at end. Area between CuP and postclaval margin light yellowish brown. Tegmen veins yellowish brown. Hind wing pale brown; veins brown. Legs and abdomen light yellowish brown to yellowish brown (Fig. 4).

***Head and thorax*.** Vertex not concave, slightly declivous; triangular areolets at lateroapical angles of head distinct; anterior margins carinate; arcus convex forward; lateral margins carinate, relatively straight, and diverging basad; posterior margin broadly concave (Figs 3, 15). Frons slightly convex in lateral view; upper margin (apex) truncate; median carina evident; lateral margin carinate, sinuately diverging to level of antennae, thence gradually incurved to suture (Fig. 16). Clypeus with distinct median and lateral carinae (Fig. 16). Rostrum just reaching trochanter of hind legs (Fig. 4). Antenna nearly cylindrical, not sunken as a depression (Figs 4, 16, 17). Ocelli separated from eyes (Fig. 17). Pronotum with 3 distinct carinae; anterior margin of disk broadly convex; posterior margin obtusely angled, concave at middle, median carina distinct, lateral carinae straight, slightly diverging rearward, attaining hind margin; lateral lobe with a small longitudinal carina between eye and tegula (Figs 3, 15). Mesonotum wider, with 3 obvious, nearly parallel carinae (Figs 3, 15).

Tegmen with costal margin slightly convex; apical margin roundly convex; distinctly concave on postclaval margin (Figs 32, 33). Stem ScP+R+MP short after basal cell before MP fork; stem ScP+R forked at ~1/3 of tegmen length, slightly before CuA fork; Vein ScP+RA with fork clearly before RP fork, with 2 terminals; branch RP with 2 terminals; Vein MP with fork clearly after CuA fork, with 3 terminals; branch CuA with 2 terminals (Figs 32, 33). Hind wing with simple ScP+RA, branch RP vein with 2 terminals, MP with 3 terminals (MP_1_, MP_2_ and MP_3+4_), vein CuA with 2 terminals, vein A2 without blind branches (Fig. 34).

***Legs*.** Post-tibiae with a lateral spine at basal 2/5. Metatibia with 7 or 8 apical teeth; rightmost apical teeth obviously larger, arrangement slightly slanting; basimetatarsomere with row of 7 apical teeth, their arrangement in arcuate; midmetarsomere with 6 or 7 apical teeth in U-shaped arrangement; metatibio-tarsal formula 8(7)–7–7 (6) (Fig. 25).

***Head*.** Vertex: A/B = 2.4. Frons: C/D = 1.4; D/E = 1.5; F/C = 0.4. Rostrum: G/H = 1.7.

***Thorax*.** Pronotum: I/B = 0.7. Mesonotum: J/I = 8.2; J/B+I = 3.4. Fore-wing: K/L = 3.1. Hind wing: M/N = 2.1.

***Male terminalia*.** Anal segment in dorsal view (Fig. 19) with apical margin roundly concave in middle, length ~1.7 times as long as width; anal style not exceeding apex of anal segment. Anal segment in lateral view (Fig. 20) with nearly right-angle bending from base to end. Pygofer in lateral view (Fig. 20) with dorsal margin distinctly shorter than ventral margin; anterior margin concave and narrowest at 1/3 its length; posterior margin near middle obviously serrated, convex. Medioventral process of pygofer in ventral view (Fig. 18) entire, apically convex, concave at base. Genital style slightly narrowing apically, with apex roundly convex, dorsal margin gives rise to 1 sharp process and 1 blunt process; inner surface near anterior margin with a slender, outwardly directed, finger-like process (Figs 18, 30, 31). Aedeagus structure relatively complex, nested (Figs 26, 27). Aedeagus with phallobase in midline deeply fissure from base to subapex, dorsal lobe in dorsal view (Fig. 26) with apical margin sharp convex; ventral periandrial lobe obviously longer than dorsal periandrial lobe. Dorsal periandrial lobe in ventral view horseshoe-shaped (Fig. 27), middle of inner margin with a longitudinal group of teeth, subapical surface in middle gives rise to a short process with its tip sharply convex, directed based; left and right lateral periandrial lobes nearly symmetrical, with tips roundly convex. Inner penis rods elongate, blade-like, curved, apically pointed (Fig. 24).

##### Etymology.

The species name is derived from the Latin word “*rotalarius*”, which refers to the medioventral process of pygofer in ventral view (Fig. 18) apically convex.

##### Host plant.

Unknown.

##### Distribution.

China (Chongqing).

## ﻿Discussion

**Figure 35.**
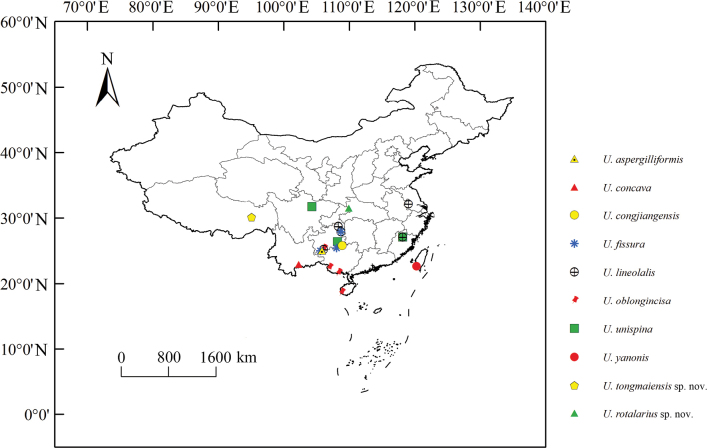
Geographic distribution of *Usana* species in China.

The genus *Usana* appears mainly distributed in the Sino-Japanese realm but six species are distributed in the tropics: *U.concava*, *U.lineolalis*, *U.oblongincisa*, *U.yanonis*, *U.abdominalis* and *U.demochares*. Among these, *U.yanonis* is the most widely distributed, since it also occurs in the Palaearctic and Oriental realms. Ten species (Fig. 35) occur in China, which appears to be a centre of endemism for the genus.

At present, the biology of *Usana* species is very poorly known. Only one host-plant, *Alangiumplatanifolium* (Sieb. et Zucc.). Harms is known for the genus, but for several species, including *U.aspergilliformis*, *U.fissura*, *U.lineolalis*, *U.oblongincisa*, and *U.unispina*, no other etho-ecological data are known.

## Supplementary Material

XML Treatment for Usana

XML Treatment for Usana tongmaiensis

XML Treatment for Usana rotalarius

## ﻿Additional information

### Author ORCIDs

Xiu-Dong Huang https://orcid.org/0000-0002-1522-4811

Lin Yang https://orcid.org/0000-0002-7841-5156

Xiang-Sheng Chen https://orcid.org/0000-0001-9801-0343

Jian-Kun Long https://orcid.org/0000-0002-4576-9919

### Data availability

All of the data that support the findings of this study are available in the main text.
